# Comparison of the full-length sequence and sub-regions of 16S rRNA gene for skin microbiome profiling

**DOI:** 10.1128/msystems.00399-24

**Published:** 2024-06-27

**Authors:** Han Zhang, Xiang Wang, Anqi Chen, Shilin Li, Ruiyang Tao, Kaiqin Chen, Ping Huang, Liliang Li, Jiang Huang, Chengtao Li, Suhua Zhang

**Affiliations:** 1 Institute of Forensic Science, Fudan University, Shanghai, China; 2 Department of Forensic Medicine, Guizhou Medical University, Guiyang, Guizhou, China; 3 Institute of Cancer Stem Cell, Cancer Center, Dalian Medical University, Dalian, Liaoning, China; 4 MOE Key Laboratory of Contemporary Anthropology, Department of Anthropology and Human Genetics, School of Life Sciences, Fudan University, Shanghai, China; 5 Shanghai Key Laboratory of Forensic Medicine, Shanghai Forensic Service Platform, Ministry of Justice, Academy of Forensic Science, Shanghai, China; 6 Key Laboratory of Cell Engineering of Guizhou Province, Clinical Stem Cell Research Institute, Affiliated Hospital of Zunyi Medical University, Zunyi, Guizhou, China; 7 Department of Forensic Medicine, School of Basic Medical Sciences, Fudan University, Shanghai, China; 8 The Key Laboratory of Environmental Pollution Monitoring and Disease Control, Ministry of Education, Guizhou Medical University, Guiyang, Guizhou, China; Southern Medical University, Guangzhou, Guandong, China

**Keywords:** full-length sequencing, *in silico* experiment, 16S variable region, skin microbiota

## Abstract

The skin microbiome plays a pivotal role in human health by providing protective and functional benefits. Furthermore, its inherent stability and individual specificity present novel forensic applications. These aspects have sparked considerable research enthusiasm among scholars across various fields. However, the selection of specific 16S rRNA hypervariable regions for skin microbiome studies is not standardized and should be validated through extensive research tailored to different research objectives and targeted bacterial taxa. Notably, third-generation sequencing (TGS) technology leverages the full discriminatory power of the 16S gene and enables more detailed and accurate microbial community analyses. Here, we conducted full-length 16S sequencing of 141 skin microbiota samples from multiple human anatomical sites using the PacBio platform. Based on this data, we generated derived 16S sub-region data through an *in silico* experiment. Comparisons between the 16S full-length and the derived variable region data revealed that the former can provide superior taxonomic resolution. However, even with full 16S gene sequencing, limitations arise in achieving 100% taxonomic resolution at the species level for skin samples. Additionally, the capability to resolve high-abundance bacteria (TOP30) at the genus level remains generally consistent across different 16S variable regions. Furthermore, the V1-V3 region offers a resolution comparable with that of full-length 16S sequences, in comparison to other hypervariable regions studied. In summary, while acknowledging the benefits of full-length 16S gene analysis, we propose the targeting of specific sub-regions as a practical choice for skin microbial research, especially when balancing the accuracy of taxonomic classification with limited sequencing resources, such as the availability of only short-read sequencing or insufficient DNA.

**IMPORTANCE:**

Skin acts as the primary barrier to human health. Considering the different microenvironments, microbial research should be conducted separately for different skin regions. Third-generation sequencing (TGS) technology can make full use of the discriminatory power of the full-length 16S gene. However, 16S sub-regions are widely used, particularly when faced with limited sequencing resources including the availability of only short-read sequencing and insufficient DNA. Comparing the 16S full-length and the derived variable region data from five different human skin sites, we confirmed the superiority of the V1-V3 region in skin microbiota analysis. We propose the targeting of specific sub-regions as a practical choice for microbial research.

## INTRODUCTION

The skin microbiome, comprising a diverse ecosystem of bacteria, fungi, and viruses, plays a pivotal role in human health by providing protective and functional benefits. Its medical applications range from diagnosing and treating skin disorders to developing personalized skincare solutions, underscoring its potential for therapeutic interventions (1–4). Research has particularly focused on the interaction between the skin microbiome and the immune system, demonstrating how microbial diversity and imbalances are associated with dermatological conditions (5–7). These insights are propelling the development of microbiome-modulating therapies. Furthermore, the inherent stability and individual specificity of the skin microbiome present novel forensic applications, where microbial traces on objects or at crime scenes may provide unique identifiers or insights into environmental interactions (8–12). However, the microbiome’s variability across different skin areas, influenced by unique microenvironmental factors such as pH, moisture levels, and sebaceous gland activity poses challenges (13–15). Therefore, a thorough taxonomic characterization of skin microbial communities, alongside the creation of uniform research protocols tailored to various skin types and DNA qualities (which are often compromised in forensic contexts), is critical for progressing in this domain. This approach will not only enhance our understanding of the skin microbiome’s role in medicine but also bolster the reliability and applicability of microbiome research in forensic science, where unique microbial signatures can be pivotal (9, 13, 16, 17).

Microbiome profiling typically involves amplicon sequencing of the bacterial 16S rRNA gene’s variable regions (V1-V9), which exhibit significant sequence diversity, facilitating bacterial identification and phylogenetic analyses (18–21). The selection of specific hypervariable regions for skin microbiome studies is not standardized but depends on the research objectives, targeted bacterial taxa, sequencing technology, and a balance between resolution, cost, and sample quality (22). The choice among the V1-V3, V3-V4, and V4 regions is pivotal for optimizing phylogenetic resolution, cost-effectiveness, and microbial diversity assessment. Although V1-V3 regions offer broad bacterial diversity coverage at a lower cost, they may not provide the finest species-level resolution. Conversely, the V3-V4 regions, preferred for Illumina sequencing due to their depth and breadth of bacterial detection, allow for distinguishing closely related taxa (23). The V4 region, compatible with universal primers and shorter read lengths, offers adequate resolution for many applications cost-effectively, albeit with reduced diversity capture compared with the combined regions. This necessitates a careful trade-off between taxonomic resolution and practical considerations, guiding researchers based on specific study requirements and constraints (16, 17).

Notably, third-generation sequencing (TGS) technology, pioneered mainly by Pacific Biosciences (PacBio) and Oxford Nanopore, has reached a level of maturity that enables the acquisition of complete or near-complete 16S rRNA gene sequences in a single, long read (24–28). This development enables more detailed and accurate microbial community analyses, extending to species and strain levels, and underscores the potential of leveraging the full discriminatory power of the 16S gene in high-throughput studies (21, 29). Although not yet universally adopted over short-read sequencing, PacBio’s full-length 16S sequencing has been instrumental in evaluating the taxonomic efficacy of individual or combined variable regions (30, 31).

In this study, we analyzed a data set of full-length bacterial 16S rRNA sequences obtained via PacBio sequencing of 141 skin samples across different human anatomical sites. By *in silico* extracting 16S sub-region data from the full data set and comparing microbial profiles and taxonomic hierarchies, we systematically assessed the impact of different sub-regions on classification efficiency. Our objective was to determine if certain sub-regions could achieve taxonomic resolutions comparable with full-length sequences, providing a valuable reference for skin microbiome researchers, especially those constrained to short-read sequencing platforms.

## MATERIALS AND METHODS

### Sample collection

In this study, we meticulously collected 141 skin microbiome specimens from 22 consenting volunteers. The collection comprised 30 intraaural skin (InaS) swabs from the external auditory canal, 31 circumaural skin (CiraS) swabs from the posterior side of the auricle and the retroauricular crease, 30 palmar skin (PaS) swabs, 20 nasal skin (NaS) swabs, and 30 oral epithelial skin (OrE) swabs. It is noteworthy that the selected participants exhibited no signs of chronic illnesses, dermatological issues, or any specific health conditions and had not undergone antibiotic treatment in the three months leading up to the sampling phase. Comprehensive information regarding the study’s objectives, methodology, and potential risks was provided to all participants, from whom written consent was secured, adhering to the ethical standards set forth in the Declaration of Helsinki (32).

The sampling procedure involved the use of sterile polyester fiber swabs, which were initially saturated in a sterile solution containing 0.15 M NaCl and 0.1% Tween 20, with the exception of the OrE swabs. For all samples, any excess solution was removed before application. Subsequently, the swabs were employed to meticulously collect samples from the skin’s surface, adopting an “S” pattern for a minimum duration of 20 seconds and incorporating a rotating movement for the collection of InaS and NaS samples, ensuring a comprehensive and uniform sample collection. The protocol and all experimental procedures received approval and were under the continuous oversight of the Ethics Committee of Fudan University, documented under approval number 2023C011, guaranteeing the adherence to ethical guidelines and integrity throughout the study’s course.

### Full-length 16S rRNA gene sequencing

Genomic DNA was extracted using the PowerSoil DNA Isolation kit. Amplification of the complete 16S rRNA gene was achieved using primers 27F (AGRGTTTGATYNTGGCTCAG) and 1492R (TASGGHTACCTTGTTASGACTT). The PCR reaction system consisted of 15 µL KOD One PCR Master Mix, 3 µL mixed PCR primers, 1.5 µL genomic DNA, and 10.5 µL nuclease-free water, with a total volume of 30 µL. PCR conditions included an initial denaturation at 95°C for 2 min, followed by 25 cycles of denaturation at 98°C for 10 s, annealing at 55°C for 30 s, extension at 72°C for 90 s, and a final extension at 72°C for 2 min. Subsequently, the PCR amplicons underwent a series of processing steps including damage repair, end repair, and adapter ligation via the SMRTbell Template Prep Kit. Purification of the PCR products was achieved using AMPure PB magnetic beads. The DNA fragment sizes were evaluated using an Agilent 2100 bioanalyzer, and concentrations were determined through Qubit fluorometry.

Prior to sequencing, the library underwent primer and polymerase attachment using the PacBio Binding kit, followed by a final purification step with AMPure PB Beads. The sequenced library, meeting all quality criteria, was analyzed on the PacBio Sequel II system by BioMarker company (Biomarker Technologies Co. Ltd., Beijing, China). Data analysis was facilitated by SMRT Link Analysis software, converting sequencer-generated BAM files into CCS sequence files, adhering to stringent parameters (minimum number of passes ≥5, minimum predicted accuracy ≥0.99). Demultiplexing of CCS sequences, based on barcode identification, was performed using lima v1.7.0. Cutadapt v1.9.1 software played a crucial role in filtering the CCS sequences by eliminating those lacking primer sequences, removing primer sequences, and selecting CCS sequences with lengths ranging from 1,200 bp to 1,650 bp.

### *In silico* extraction of 16S sub-regions from full-length sequencing data

**Fig 1:**
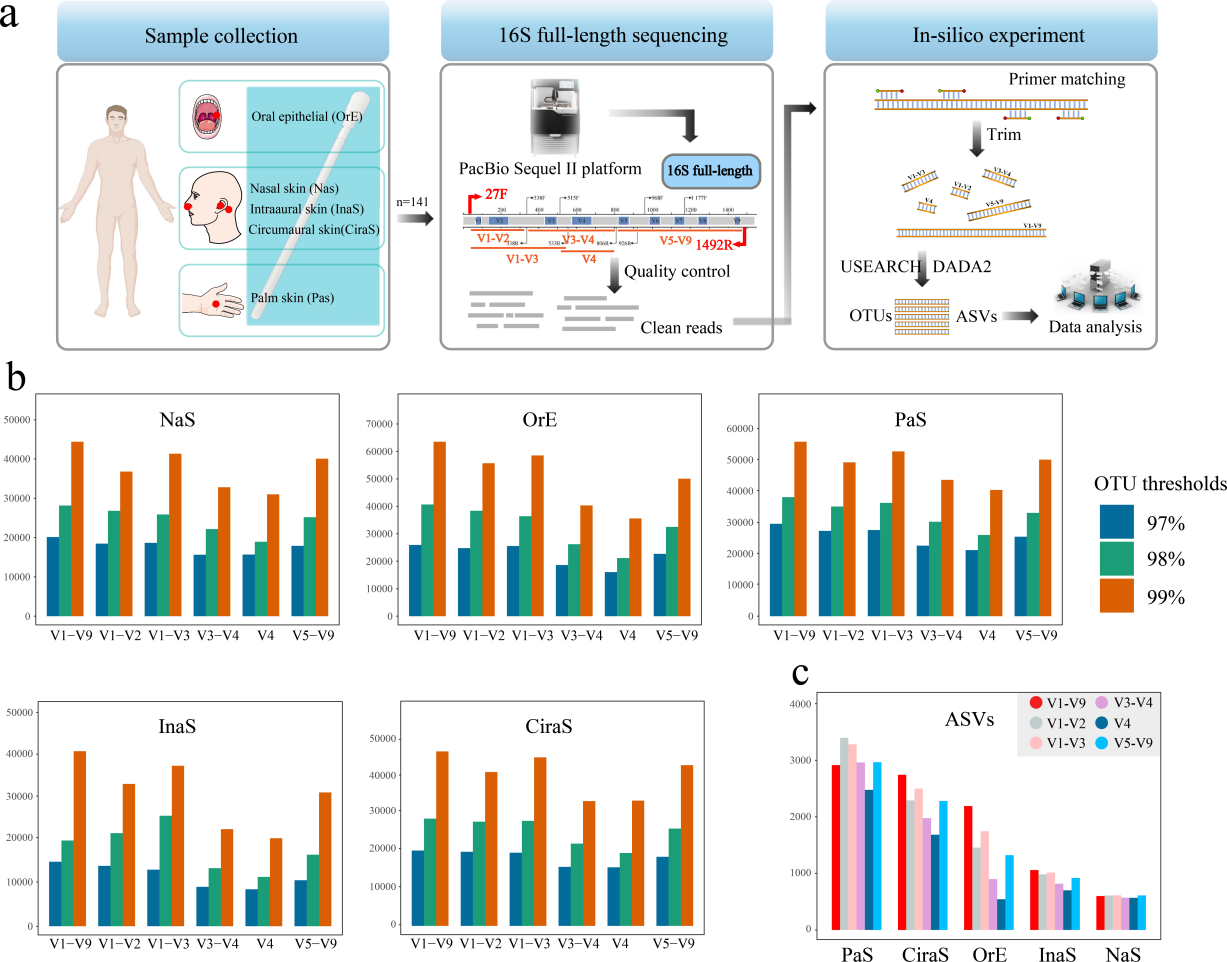
Overview of methodology and results for microbial community analysis in skin samples. (**a**) Schematic representation of the study’s workflow, encompassing sample collection from various skin sites, experimental procedures, and the analytical pipeline employed for data interpretation. (**b**) Distribution of Operational Taxonomic Units (OTUs) identified in different 16S rRNA gene regions across five skin sample types: intraaural skin (InaS), circumaural skin (CirS), palm skin (PaS), nasal skin (NaS), and oral epithelial skin (OrE). (**c**) Comparative analysis of Amplicon Sequence Variants (ASVs) across different 16S rRNA gene regions for the InaS, CirS, PaS, NaS, and OrE.

In the computational analysis of 16S rRNA gene sequences, the delineation of the V1-V9 region represents the entirety of the full-length 16S rRNA gene for the purposes of this investigation. Distinct sub-regions of the 16S rRNA gene, including V1-V2, V1-V3, V3-V4, V4, and V5-V9, were meticulously extracted from the comprehensive full-length sequences. This extraction process was guided by the specific locations of PCR primer binding sites, which are routinely utilized in microbiome research endeavors (Fig. 1a). The extraction methodology is detailed in the following procedural steps:

1. Primer pair identification: Commence by cataloging all possible primer pair combinations located in the conserved regions flanking the target variable regions. These primer pairs are then aligned with the full-length 16S rRNA gene sequence. Subsequently, sequences encapsulated by these primer pairs are extracted and preserved as Fastq format files.
2. Tolerance setting for primer matching: Implement a tolerance threshold that allows for up to four base mismatches within both the forward and reverse primers during the matching process. This flexibility aims to accommodate minor sequence variations, thereby ensuring a comprehensive capture of the target sequences.
3. Read count verification: Post-extraction, perform a quantitative assessment of the derived reads to verify that the capture efficiency surpasses the 98% threshold. This step is critical for confirming the robustness and reliability of the primer matching and sequence extraction process.
4. Accuracy assessment through random selection: To validate the fidelity of the extracted variable region sequences, a random subset of these sequences is selected and compared against the original full-length 16S sequences. This comparative analysis serves to confirm the precision of the sequence capture and the integrity of the variable regions extracted.

Refer to Table S1 for detailed primer specifications utilized in this study.

### Data analyses

The data analysis framework encompassed a series of sophisticated bioinformatics procedures designed to refine and interpret the 16S rRNA gene sequencing data. Initially, low-quality sequences were rigorously excluded, and chimeric sequences were identified and excised employing the default settings of Cutadapt software. This pre-processing step ensured that only high-quality, authentic sequences were advanced for further analysis.

Subsequent to the quality filtration, representative sequences for each Amplicon Sequence Variant (ASV) were delineated utilizing QIIME2 software (v1.9.1) (33). This delineation was conducted based on both the full-length 16S rRNA gene sequences and their respective variable regions. The ASVs identified were then subjected to annotation and comparison against the SILVA database (version 138) using the q2-feature-classifier plugin, facilitating a precise taxonomic classification (34).

OTUs were generated at different sequence similarity thresholds (97%, 98%, and 99%) through the application of USEARCH software (v11) (35). Subsequently, each representative OTU underwent genus-level annotation, employing the Ribosomal Database Project (RDP) classifier (v2.13), with a confidence threshold set at 0.8.

Diversity within the microbial communities was quantified through alpha-diversity (Shannon index) and beta-diversity (Bray-Curtis distances) metrics (36), calculated using QIIME2 software. To visualize the microbial community structure and diversity, Principal Coordinate Analysis (PCoA), Non-Metric Multidimensional Scaling (NMDS), and Unweighted Pair Group Method with Arithmetic Mean (UPGMA) were constructed based on Bray-Curtis dissimilarity matrices. These visual representations were generated using the ggplot2 package within the R programming environment.

For the identification of statistically significant biomarkers across different sample groups, Linear Discriminant Analysis Effect Size (LEfSe) analysis was conducted (37), setting the Linear Discriminant Analysis (LDA) score threshold to 4. The random forest machine learning methods were implemented with R script to predict the origin of the samples (38). Additionally, sequence variation within the primer-binding sites of the 16S rRNA gene was meticulously analyzed using custom in-house R scripts, offering insights into the primer specificity and efficiency across diverse microbial taxa.

## RESULTS

### Characterization of 16s rRNA full-length sequencing data

The comprehensive sequencing of the 16S rRNA gene from 141 skin microbiota samples yielded a substantial data set of 1,345,711 raw reads, with individual sample reads varying significantly, from a minimum of 4,314 to a maximum of 14,658 reads. Following the implementation of a rigorous denoising process, integral to the quality control measures, the data set was refined to 1,015,343 clean tags. These clean tags exhibited a broad range in quantity per sample, spanning from 984 to 14,064, reflecting the effectiveness of the denoising process which varied widely across samples, from as low as 13.04% to as high as 99.28%, with an overall average effectiveness of 71.61%. This wide range in data quality and quantity underscores the heterogeneity inherent in biological sampling and the subsequent necessity for stringent quality control to ensure the reliability of downstream analyses.

The diversity within these samples was further evidenced by the number of ASVs detected, ranging from a minimal count of 10 to a substantial count of 808 ASVs per sample. Collectively, a total of 69,616 ASVs were identified across all samples and were retained for further analytical scrutiny. This data set of ASVs serves as a testament to the microbial diversity present across the sampled skin sites and provides a foundation for the subsequent analyses aimed at elucidating the complex microbial ecosystems of the human skin.

### Taxonomic classification

Our microbial community analysis employed two distinct clustering methodologies: OTUs and ASVs. Implementing different clustering thresholds (97%, 98%, and 99%) led to variations in OTU numbers, which were influenced by different variable regions within the five sample types examined, as depicted in Fig. 1b. Notably, analyses using full-length 16S rRNA sequences identified the most OTUs. Conversely, the V3-V4 and V4 regions yielded fewer OTUs, whereas the V1-V3 and V1-V9 regions demonstrated comparable OTU counts. The distribution of ASVs among the sample types was ranked as follows: PaS >CiraS > OrE >InaS > NaS, as shown in Fig. 1c. Within the same sample category, the regions V1-V9, V1-V3, and V5-V9 revealed a higher abundance of ASVs, in contrast to the reduced ASV counts found in the V3-V4 and V4 regions, as shown in the CiraS, InaS, and OrE samples. Of note, the V4 region contained the smallest number of ASVs in OrE samples. However, the PaS samples exhibited a significantly different trend. A notably higher number of ASVs detected in the V1-V2 and V1-V3 regions of PaS samples compared with the V1-V9 region. NaS samples manifested the least variability in ASV numbers across different sub-regions.

**Fig 2:**
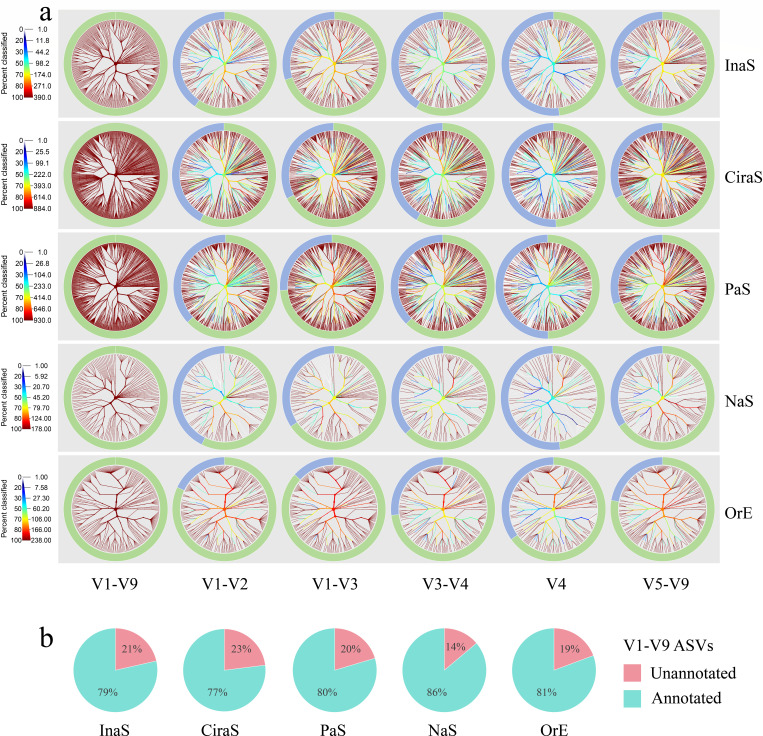
Comparative analysis of phylogenetic trees and taxonomic annotation across hypervariable regions. ASVs are annotated at the species level in the V1-V9 region as the standard data set. This figure features phylogenetic trees paired with donut pie charts, showcasing bacteria at the species level across various hypervariable regions of 16S rRNA genes from five types of skin samples studied. (**a**) In the phylogenetic trees constructed based on unique bacteria (V1-V9), the same tree is provided for each sub-region. The color of each branch indicates the proportion of bacteria within each clade that are identified to species level. In the donut pie charts, green segments denote the unique bacteria annotated at the species level, and blue segments represent those bacteria unannotated to the species level. (**b**) ASVs from the V1-V9 region that annotated or could not be annotated at the species level, across five types of samples studied.

Subsequently, we employed ASV sequence information to achieve taxonomic resolution of bacterial communities. Here, we take ASV sequences annotated at the species level in the V1-V9 region as the standard data set to compare the discrimination ability at the species level of different 16S rRNA hypervariable regions. This process is visualized in Fig. 2a, where phylogenetic trees have been constructed for unique bacteria at the species level. Here, we need to emphasize that for the annotation at the species level of different hypervariable region sequences extracted from the V1-V9 sequences, they must be completely consistent with the corresponding V1-V9 sequence annotation information. Otherwise, they will not be included, even if they are also annotated at the species level. Our findings reveal a notable trend: the V1-V9 region contains a higher number of annotated species, resulting in a markedly more dense circular branching pattern deeper red in the phylogenetic tree. Following that, the V1-V3 and V5-V9 regions, with the V4 region being the least dense, result in branches being displayed in a deeper blue. However, this pattern of annotation was consistent across the other 16S rRNA hypervariable regions for the same type of samples, with no significant differences in phylogenetic tree complexity. The outer donut pie charts, highlighted in green, further illustrate this difference. They show a progressive decrease in the number of species from the V1-V9 region to the V4 region, with intermediate counts in the V1-V3, V5-V9, V1-V2, and V3-V4 regions. Moreover, when examining the comprehensive 16S sequences, the phylogenetic trees for PaS and CiraS samples reveal a higher density of branches. This suggests a greater diversity of bacteria within these samples at the species level, indicating a further research on bacterial taxa needs to be fully explored or documented. However, reviewing the ASVs generated from the entirety of the sequencing efforts, even based on the full-length 16S sequence information, there is still a certain proportion of sequences (14%-23%) that cannot be annotated at the species level. It is worth noting that this proportion varies with the type of sample (Fig. 2b).

### Comparisons of microbial community structure and diversity analysis

**Fig 3:**
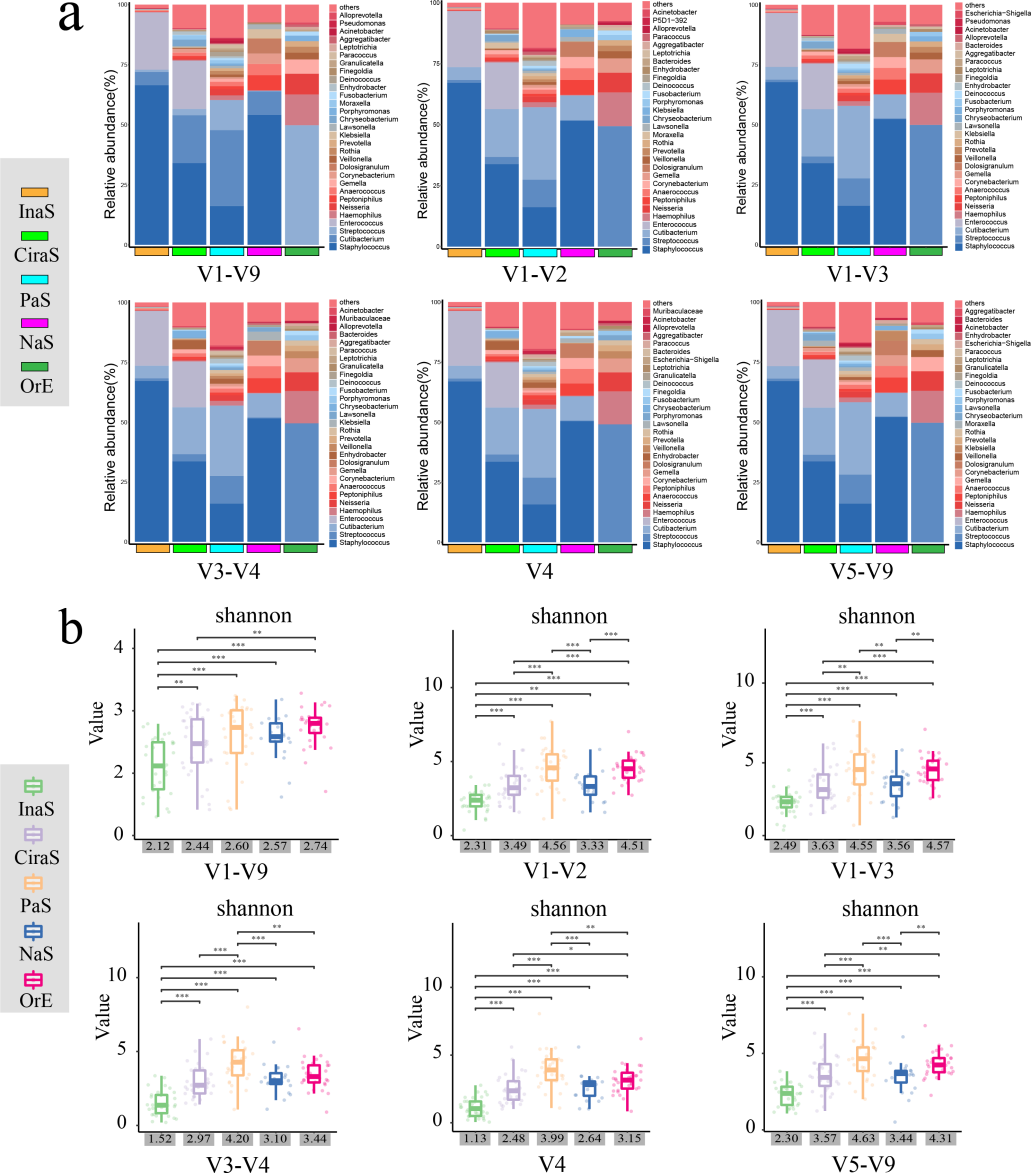
Overview of microbial community composition and alpha diversity metrics. (**a**) Depicts the distribution of the top 30 most abundant bacterial genera across different 16S rRNA gene regions within the five skin sample types. The “others” category represents all bacteria of lower abundance. (**b**) Features a box plot detailing the Shannon diversity index across each 16S region, with the x-axis displaying the average Shannon index values for the various sample types. Statistical significance is denoted as follows: **P < 0.01, ***P < 0.001, determined by *t*-tests.

We created stacked bar charts at both the genus and species levels for the top 30 bacteria across different sub-regions of various sample types (Fig. 3a; Fig. S1). In these visual representations, distinct colors denote different bacterial taxa, and the height of each colored segment indicates the proportion of a given bacterium’s abundance relative to the total bacterial abundance observed. The category labeled “others” represents the relative abundance of bacteria that did not rank among the top 30.

Upon meticulous examination and comparison of these charts, we observed that at the genus level (as shown in Fig. 3a), the microbial community structures within the same sample types appeared remarkably consistent across the various regions, including the V1-V9 region. These structures exhibited only subtle variations in their relative abundances. Furthermore, when we arranged the abundance values, it became apparent that the top three genera within the different hypervariable regions of the same sample types were exactly the same, underscoring a high degree of consistency at the genus level.

Conversely, at the species level (as shown in Fig. S1), even a straightforward visual assessment reveals significant differences in microbial community compositions across the different 16S sub-regions within the same sample types. These differences were not only in terms of the relative abundances but also in their bacterial species present. Notably, when comparing the top 30 bacterial species by abundance across the hypervariable regions with the V1-V9 region, the V5-V9 region showed the highest similarity, with 28 species in common. This was followed by the V1-V3 region, which shared 25 species, whereas the V4 region demonstrated the least similarity, sharing only nine species.

To further our understanding of the diversity within microbial communities, we calculated the Shannon index, a metric that quantifies both species richness and evenness. The findings, illustrated in Fig. 3b through boxplots, showed a uniform trend across various sub-regions and sample types. Specifically, samples labeled PaS presented the highest Shannon index values, indicating the most diverse microbial community, whereas InaS displayed the lowest values, suggesting a less diverse community composition.

Interestingly, when comparing samples of the same type across different 16S rRNA gene regions, the highest average Shannon index was not found in the V1-V9 region, but rather in the V1-V3 region. Conversely, the V4 region was associated with the lowest diversity index. This indicates that the V1-V3 region might be more reflective of microbial diversity in skin samples.

Through *t*-test analysis, significant differences in the Shannon index values among the different sample types were identified, with statistical significance denoted by asterisks (*P* < 0.05). This suggests that the diversity of microbial communities varies notably across different environmental or biological contexts. However, it is noteworthy that the differences in species richness indicated by the Shannon index were less marked in the V1-V9 region compared with other regions. This pattern was particularly evident among the PaS, CiraS, NaS, and OrE samples.

### Comparative analyses among five distinct skin sample types

**Fig 4:**
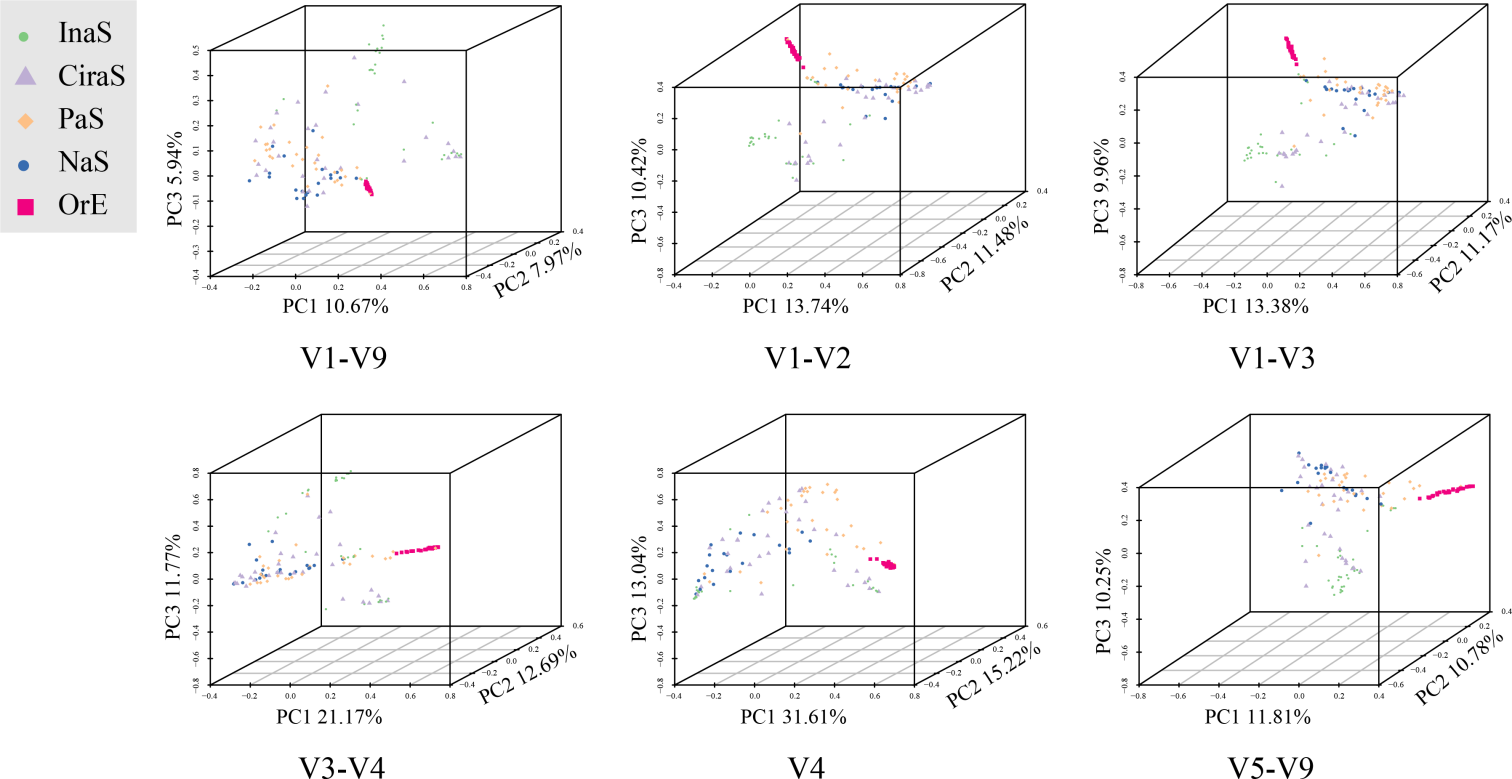
Three-dimensional principal coordinate analysis (PCoA) plot for different types of skin samples using the Bray-Curtis distance matrix.

PCoA and NMDS were meticulously conducted to explore the microbial community structures within five different skin sample types, with findings presented in both two-dimensional and three-dimensional scatter plots (refer to Fig. 4; Fig. S2). The analyses aimed to discern patterns of similarity and dissimilarity based on variations within the 16S rRNA regions across the sample types. Notably, the OrE samples, represented in red within the scatter plots, demonstrated a pronounced clustering effect, distinctly segregating from the other sample types. This clear demarcation underscores the unique microbial signature of the OrE samples. The InaS samples, marked in green, also showed a coherent clustering but to a lesser extent, suggesting a distinguishable yet closely related microbial community composition in comparison to other sample types. Conversely, the samples associated with CiraS, PaS, and NaS exhibited considerable overlap in their clustering patterns. This convergence indicates a significant challenge in differentiating among these sample types based solely on their microbial 16S rRNA profiles. The intermingling of the CiraS, PaS, and NaS samples highlights the complex interplay and potential similarities in the microbial communities present in these diverse environmental contexts.

The construction of a phylogenetic tree, utilizing the UPGMA algorithm, corroborated the insights gained from the earlier PCoA, as illustrated in Fig. S3. This phylogenetic analysis further substantiates the lack of significant differences in the clustering of microbial communities among the varied 16S regions, aligning with the PCoA findings. Specifically, the OrE samples, highlighted in red, exhibited the most coherent and distinct clustering within the phylogenetic tree. In stark contrast, the CiraS, represented in purple, displayed the least effective clustering. This alignment between the UPGMA-based phylogenetic tree and the PCoA results highlights the complexities of microbial community compositions that transcend simple classification based on 16S rRNA gene regions. The detailed analysis reveals both the distinctiveness and the subtleties of microbial associations across diverse skin samples, underscoring the intricate relationships within microbial ecosystems.

### Identification of bacterial biomarkers across diverse skin types and construction of random forest prediction model

**Fig 5:**
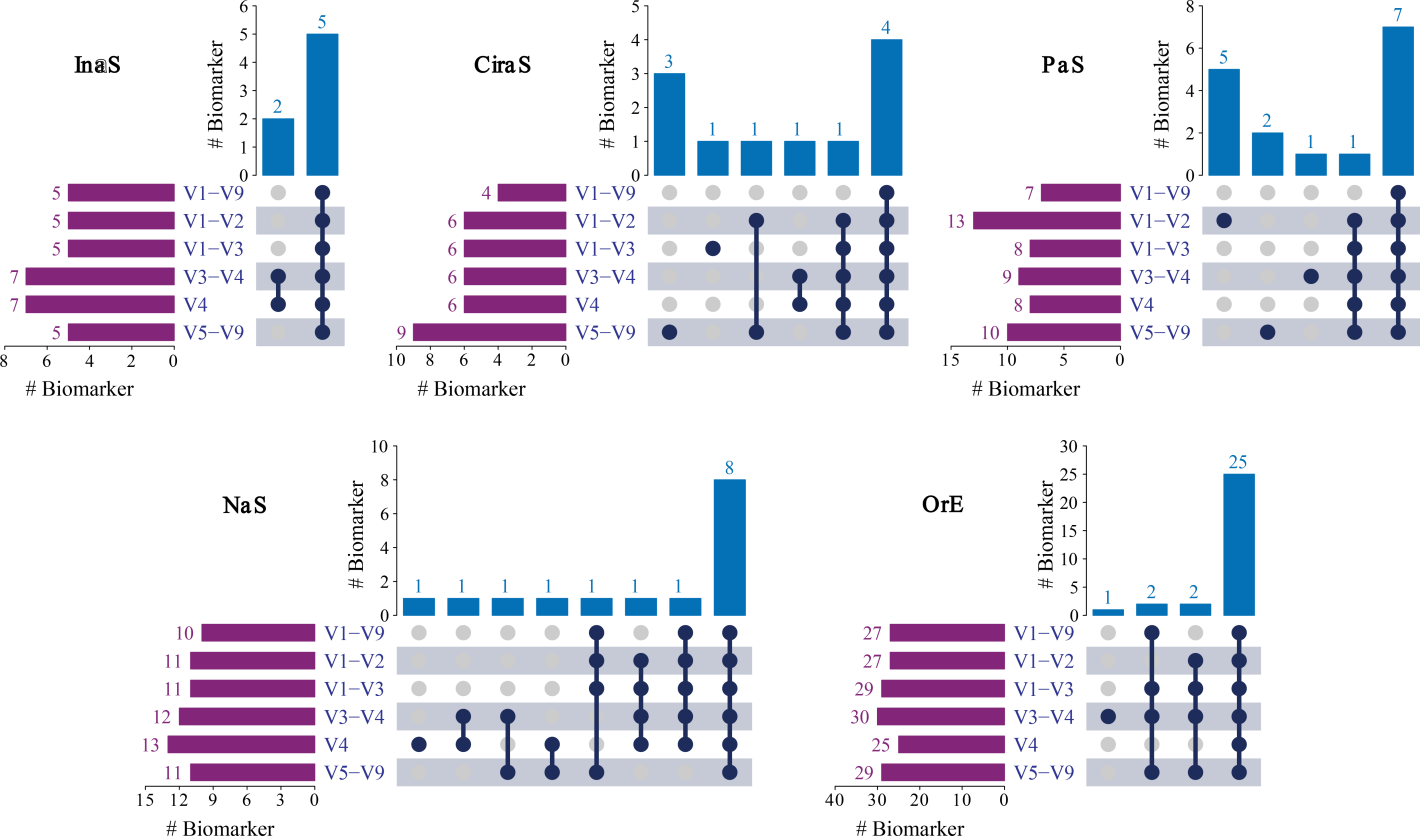
Upset plot showing the shared microbial biomarkers between the individual 16S regions from five types of skin samples studied.

We employed the LEfSe method to identify microbial biomarkers among the five types of samples, with a stringent threshold for significance (LDA score >4, *P* < 0.05) (Table S2). Overall, the number of potential biomarkers selected based on the V1-V9 sequence is less than that from other 16S sub-regions (Fig. 5). The majority of biomarkers identified from V1-V9 region were included within the set of bacterial biomarkers within the sub-regions. Additionally, the extra biomarkers identified in other 16S sub-regions compared with the V1-V9 region tend to exhibit lower LDA scores, such as *Enterococcus* and *Enterococcaceae* in InaS sample; *Chryseobacterium*, *Enhydrobacter,* and *Moraxella* in CiraS sample; *Micrococcales* in PaS sample; *Roseimarinus* and *Klebsiella* in NaS sample; and *Rothia* and *Micrococcaceae* in OrE sample. It is noteworthy that the biomarker *Clostridia* at the class level, identified in the V1-V2, V1-V3, V3-V4, and V4 regions in the OrE sample, was not detected in the V1-V9 region, and it has the highest LDA value.

To infer the origin of the samples, we constructed a random forest model. The performance of the model was evaluated using four parameters: overall accuracy, precision, recall, and F1 score (Table 1). The overall accuracy of random forest models built using various 16S regions exhibited slight variation, ranging from 70% to 85%. Unexpectedly, the model in the V1-V9 region showed the worst prediction accuracy, whereas the V4 region achieved the highest accuracy. Among these, the prediction accuracy for OrE samples was the highest, reaching 100%, followed by NaS samples based on the F1 score. For other sample types, the prediction accuracy of each variable region followed this order: PaS >InaS > CiraS, while for the V1-V9 region, it was InaS > PaS > CiraS.

**TABLE 1:** Overall accuracy, precision, recall (sensitivity), and F1 score of the random forest classifier for different 16S regions across the five types of skin samples

		InaS (%)	CiraS (%)	PaS (%)	NaS (%)	OrE (%)
V1-V9	Random Forest (Overall accuracy 73.81%)					
	Precision	57.14	66.67	71.43	83.33	100.00
	Recall	88.89	44.44	55.56	83.33	100.00
	F-1 Score	69.56	53.33	62.50	83.33	100.00
V1-V2	Random Forest (Overall accuracy 78.57%)					
	Precision	57.14	66.67	100.00	100.00	100.00
	Recall	88.89	66.67	66.67	66.67	100.00
	F-1 Score	69.56	66.67	80.00	80.00	100.00
V1-V3	Random Forest (Overall accuracy 80.95%)					
	Precision	57.14	71.43	100.00	100.00	100.00
	Recall	88.89	55.56	77.78	83.33	100.00
	F-1 Score	69.56	62.50	87.50	90.91	100.00
V3-V4	Random Forest (Overall accuracy 80.95%)					
	Precision	66.67	62.50	100.00	85.71	100.00
	Recall	88.89	55.56	66.67	100.00	100.00
	F-1 Score	76.19	58.83	80.00	92.31	100.00
V4	Random Forest (Overall accuracy 83.33%)					
	Precision	66.67	75.00	100.00	85.71	100.00
	Recall	88.89	66.67	66.67	100.00	100.00
	F-1 Score	76.19	70.59	80.00	92.31	100.00
V5-V9	Random Forest (Overall accuracy 80.95%)					
	Precision	61.54	66.67	100.00	100.00	100.00
	Recall	88.89	66.67	66.67	83.33	100.00
	F-1 Score	72.73	66.67	80.00	90.91	100.00

Additionally, we calculated the mean decrease Gini (MDG) values and selected 30 important species at the genus level, as shown in Table S3. The results showed that the important species in various sub-regions were not much different from those in the V1-V9 region, and there were only no more than two species differences. It is worth noting that the important species in the V5-V9 region were completely identical to those in the V1-V9 region.

### Sequence variation of 16S rRNA gene primer-binding sites

**Fig 6:**
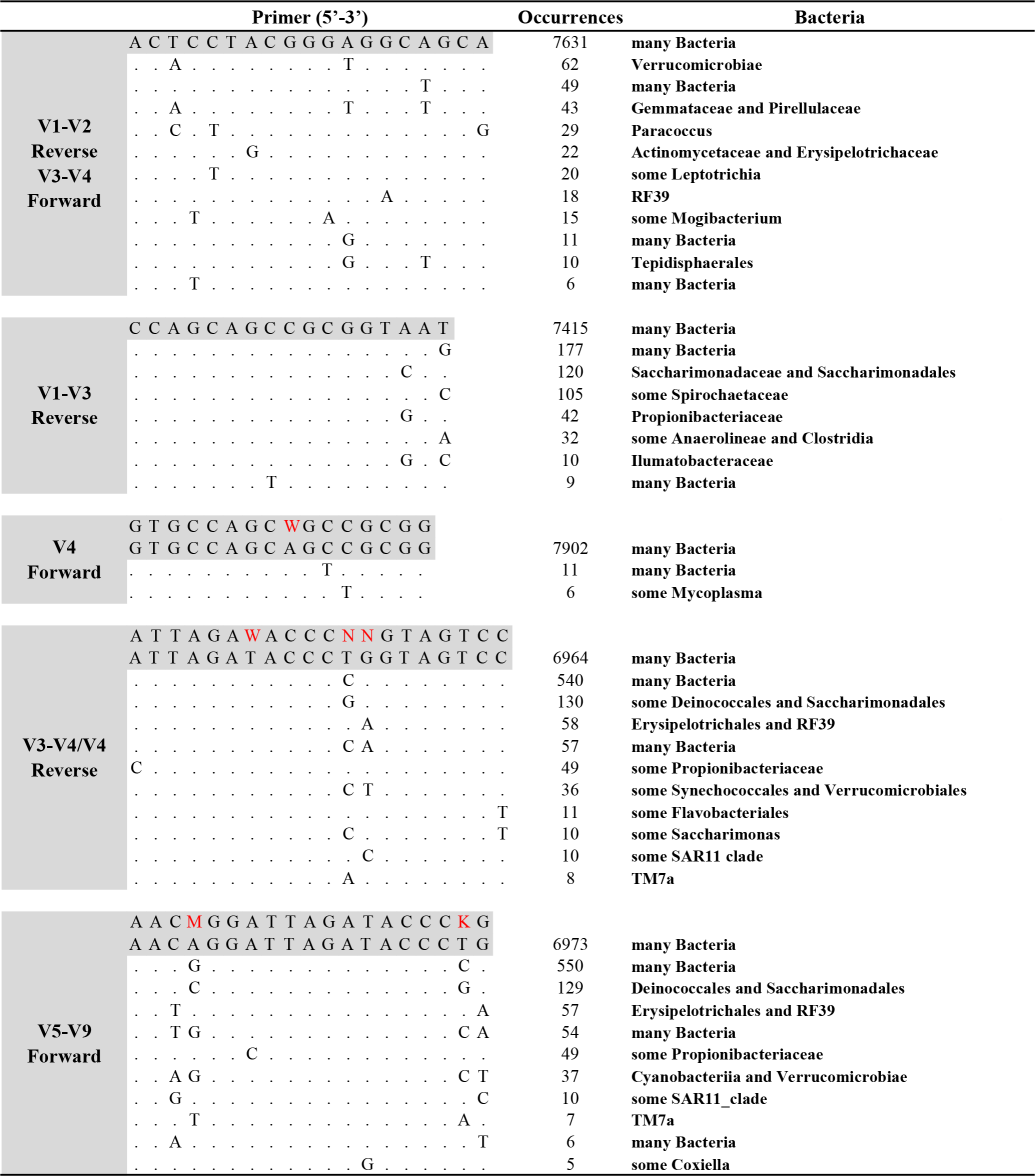
Sequence variation of 16S rRNA gene primer-binding sites. Nucleotide sites identical to the most common consensus sequence (the first listed) are represented as dots. The degenerate primer sequences are shaded gray, and the main variation sites are highlighted in red.

In our study, we conducted an in-depth statistical analysis to assess sequence variability at the primer binding sites. Taking the NaS sample as an example, we combined the full-length 16S sequence data from all individuals, eliminated duplicate sequences, and curated a set of “unique” sequences. We then extracted subsets of sequences corresponding to each of the primer binding sites used in our analyses. Figure 6 presents the prevalent sequence variations and identifies the predominant bacterial groups associated with these variations. To ensure statistical relevance, we excluded sequence variations with fewer than five reads, operating under the premise that variations constituting less than 0.1% of the total may lack authenticity or statistical significance. Our findings indicate that primer binding sites exhibited varying numbers of base variations, many of which did not fall within the range of degenerate primers.

A particularly noteworthy observation is that specific bacterial groups were associated with unique primer variants. Among the primers evaluated, the forward primer designed to target the V4 region displayed minimal variation. However, challenges were identified with other primer sets: notably, the commonly employed reverse degenerate primer for the V4 region was found to mismatch with sequences from the *Propionibacteriaceae*, *Flavobacteriales,* and *Saccharimonas*. This mismatch may hinder the amplification and subsequent detection of these groups. Similarly, the forward primer for the V5-V9 region demonstrated potential mismatches at two positions for taxa such as *Erysipelotrichales*, *RF39, Cyanobacteriia,* and *Verrucomicrobiae*, potentially reducing the sensitivity of detection for these microbial groups. The forward primer for the V3-V4 region and reverse primer for the V1-V2 region demonstrated potential mismatches at three positions such as *Gemmataceae*, *Pirellulaceae,* and *Paracoccus*.

## DISCUSSION

The landscape of human skin microbiology research is rapidly evolving, with a pronounced shift toward extensive sample collection, cohort studies, and practical applications spanning various sectors including medicine, environmental science, and forensics, as highlighted in references (39–41). The full-length 16S rRNA gene sequencing marks a transformative advancement in microbial research, facilitating an unparalleled taxonomic resolution of microbial community compositions, as noted in references (21, 39). Despite its advantages, the adoption of full-length 16S rRNA gene sequencing is hampered by several challenges including the selection of sequencing platforms, the technical complexity of the methodologies, stringent requirements for DNA integrity and content, and the associated high costs. In this context, opting for targeted sequencing of specific 16S sub-regions using second-generation high-throughput sequencing technologies emerges as a pragmatic approach to satisfy the foundational requirements of both research and practical applications (42).

This study leverages full-length 16S sequencing data derived from microbial communities across various human skin sites to systematically assess the classification efficiency of different 16S sub-regions. It unequivocally demonstrates that full-length 16S sequences offer a more comprehensive genetic insight into microbial communities and enable a higher resolution of microbial community profiling. Furthermore, our findings affirm that the V1-V3 region is particularly well-suited for skin microbiome profiling, corroborating previous research findings (22). Notably, the resolution provided by the V1-V3 region more closely approximates that of full-length 16S sequencing compared with other hypervariable regions, underscoring its suitability and effectiveness for detailed microbial community analysis in skin microbiome studies (21).

Our study has elucidated that full-length 16S ribosomal RNA (rRNA) gene sequencing possesses a superior discriminatory capability compared with targeted sequencing of specific sub-regions. Despite this advantage, a significant number of ASVs within the V1-V9 region remain unannotated at the species level (14%-23%), highlighting a limitation in the resolution of skin microbial identification. Here, we propose several possibilities: (i) balance between conservation and variability: the 16S rRNA gene contains highly conserved and variable regions; even with full-length sequencing, the conservation of these regions may not provide sufficient resolution to distinguish genetically very close species (43). (ii) Intraspecies genetic variation: Even within the same species, different strains may have variations in the 16S rRNA gene sequence. This intraspecies variation can sometimes make species-level identification challenging (44). (iii) Technical and methodological limitations: sequencing errors and primer bias can affect the quality and accuracy of sequencing data. Additionally, the accuracy of the bioinformatics tools and databases used can impact the final species identification (16). (iv) Incompleteness of databases and reference sequences: many microbes have not yet been cultured and sequenced, meaning that even with full-length 16S rRNA sequencing, it might not be possible to accurately identify all species (45). (v) Ecological and evolutionary factors: gene horizontal transfer and recombination events among microbial species can cause differences in the 16S rRNA gene sequence, which may mislead species identification and analysis (46).

Furthermore, our comparative analysis between ASV and OTU clustering methodologies yielded not entirely consistent results, which align with expectations, given their fundamentally different algorithmic bases, as referenced in (47). OTU clustering groups sequences that exhibit less than a predetermined percentage of dissimilarity, commonly set at 3%, potentially leading to the amalgamation of similar yet taxonomically distinct bacteria within the same OTU, as discussed in references (48, 49). Conversely, ASVs, generated through the divisive amplicon denoising algorithm of DADA2 (50), offer a much finer resolution, distinguishing sequences based on single-nucleotide differences. This precision has contributed to the growing popularity of ASVs for microbial community analysis (49). Given these distinctions, our research predominantly focused on the enumeration and analysis of ASVs.

Notably, PaS and CiraS samples harbored a substantial number of bacterial species that could not be definitively classified at the species level. We hypothesize that this phenomenon may be attributed to the direct and frequent interaction of PaS and CiraS samples with the external environment (51, 52). Such interactions facilitate transient colonization by a wide array of environmental microbes, thereby enriching the microbial diversity within these samples but also increasing the prevalence of taxa represented by lower abundance. This observation underscores the complex and dynamic nature of microbial communities, particularly in contexts with high environmental exposure, and highlights the challenges in achieving comprehensive microbial identification and classification (53).

Differences in the taxonomic resolution of various 16S variable regions in analyzing bacterial community composition and diversity are minimal for the same type of samples. Although there are significant differences at the species level, we contend that this does not accurately reflect the actual situation, as our previous analyses have shown limited resolution even with full-length 16S sequencing. This seems to be supported in part by the high proportion of “others” at the species level. One possibility is that more bacteria were not annotated at the species level. Thus, caution is required when using 16S sequences to study skin microbial communities at the species level, we recommend that research should be conducted at the genus level or higher or more recommended metagenomic sequencing analysis (21, 42, 54).

The PCoA, NMDS, and UPGMA cluster analyses adopted in this study consistently demonstrated that clustering of the skin samples based on different 16S sub-regions did not show obvious differences. Judging from the clustering results of the OrE samples, we believe that the effectiveness of differentiation is more dependent on whether the microbial community composition characteristics of the samples themselves are significant. When collecting samples, it was inevitable that a certain amount of saliva was mixed into the OrE samples. The microbial community composition of saliva is significantly different from that of skin samples (55), leading to the significant difference between OrE and other sample types, manifested as pronounced self-clustering and clear separation from other samples.

The microbial biomarkers with high LDA values are generally consistent across the different 16S sub-regions including the V1-V9 region. However, the sub-region has more unique microbial biomarkers with lower LDA values, especially for PaS and CiraS samples with high environmental exposure, and microbial community diversity. We posit that the main reason for this phenomenon is that 16S hypervariable regions sequencing reads contain limited sequence variation, as well as inherent preferences in primer binding and microbial annotation, which introduce systematic errors during the analysis of different types of samples, resulting in varying outcomes for the same type of samples analyzed in different 16S hypervariable regions (14, 16).

Despite numerous analyses in this study revealing that the V4 region performed worst in classifying skin bacterial communities, it exhibited the highest accuracy in predicting the sample origin based on the random forest algorithm. This is indeed difficult to explain reasonably. We speculate that the excessively low genetic information content in the V4 region, leading to poor analytical ability and higher error rate of the V4 region may artificially increase the differences in microbial communities between different types of samples, thereby making successful predictions easier (30, 56). Moreover, when we selected the TOP 30 important bacteria based on the MDG values, there were no significant differences in the various variable regions. This implies that there may be other factors causing differences in the predictive ability of the random forest model, such as low MDG values or low abundance bacteria (57).

Building upon our findings and grounded speculations, our investigation extended to the sequence variation at the primer-binding sites of the 16S rRNA gene, which reaffirmed the variability inherent to these sites across different hypervariable regions. Importantly, our statistical analysis revealed that certain sequence variations displayed a predilection for specific bacterial taxa. This finding partially elucidates the differences observed among the various 16S hypervariable regions and their comparison to the V1-V9 regions, offering a tangible explanation for the differential outcomes noted in microbial community analyses. The occurrence of base sequence variations at primer-binding sites represents a facet of biological evolution, a phenomenon that necessitates a more nuanced understanding. Consequently, there is a pressing need for more exhaustive and detailed research efforts aimed at cataloging these variations. Such efforts should strive to identify patterns within the sequence variability of 16S rRNA, enabling the selection of the most appropriate hypervariable regions for specific research endeavors. These findings highlight the critical importance of primer selection and design in the accurate representation and analysis of microbial communities, underscoring the need for careful consideration of primer specificity and the potential for sequence variation to impact microbial diversity studies.

### Conclusions

Our comprehensive analysis underscores that full-length 16S rRNA gene sequencing offers superior taxonomic resolution for delineating the composition of the skin microbiome over methods that target specific variable regions. This enhanced resolution facilitates a more accurate and detailed understanding of microbial communities present on the skin. Interestingly, our findings also highlight the V1-V3 region’s comparably high discriminatory power, which rivals that of the entire 16S gene. This observation is particularly significant as it provides valuable insight for researchers when selecting the most appropriate 16S regions for their studies, especially in contexts where sequencing resources may be limited or when DNA quality is insufficient for full-length 16S rRNA gene sequencing.

The equivalence in the discriminatory potential between the V1-V3 region and the full 16S rRNA gene sequencing is a critical insight for fields such as forensic microbiology, where precise taxonomic resolution is paramount for the accurate identification of microbial evidence. This knowledge allows for more informed decision-making regarding the selection of 16S rRNA gene regions for sequencing efforts, balancing the need for detailed taxonomic resolution with the practical considerations of sequencing costs and technical complexity.

Moreover, these findings serve as a vital reference for the broader research community, guiding the selection of sequencing strategies that best align with the objectives and constraints of various studies. By informing the choice of targeted 16S regions based on their demonstrated taxonomic resolution capabilities, our study contributes to the optimization of microbial research methodologies across diverse fields, enhancing the accuracy and efficiency of different skin microbial community analyses.

## Data Availability

The raw sequencing data from this study have been deposited in the Genome Sequence Archive (GSA), Chinese Academy of Sciences (GSA: CRA015299) that are publicly accessible at https://ngdc.cncb.ac.cn/gsa.

## SUPPLEMENTAL MATERIAL

Fig. S1 msystems.00399-24-s0001.tif Distribution of the top 30 high-abundance bacteria at the species level for various 16S regions.

Fig. S2 msystems.00399-24-s0002.tif Two-dimensional NMDS plot for different types of skin samples.

Fig. S3 msystems.00399-24-s0003.tif Phylogram tree constructed from different types of skin samples.

Supplemental legends msystems.00399-24-s0004.docx Legends for Figures S1 to S3 and Tables S1 to S3.

Table S1 msystems.00399-24-s0005.xlsx Primer pairs targeting regions of the 16S rRNA gene.

Table S2 msystems.00399-24-s0006.xlsx The microbial biomarkers and LDA values for different 16S regions across the five types of skin samples.

Table S3 msystems.00399-24-s0007.xlsx The top 30 importance species and MDG values.

ASM does not own the copyrights to Supplemental Material that may be linked to, or accessed through, an article. The authors have granted ASM a non-exclusive, world-wide license to publish the Supplemental Material files. Please contact the corresponding author directly for reuse.
