# Supplementary figures and images for: Comparison of the full-length sequence and sub-regions of 16S rRNA gene for skin microbiome profiling
Source: mSystems. 2024 Jun 27;9(7):e00399-24. doi: 10.1128/msystems.00399-24 (PMC11264597; doi:10.1128/msystems.00399-24)

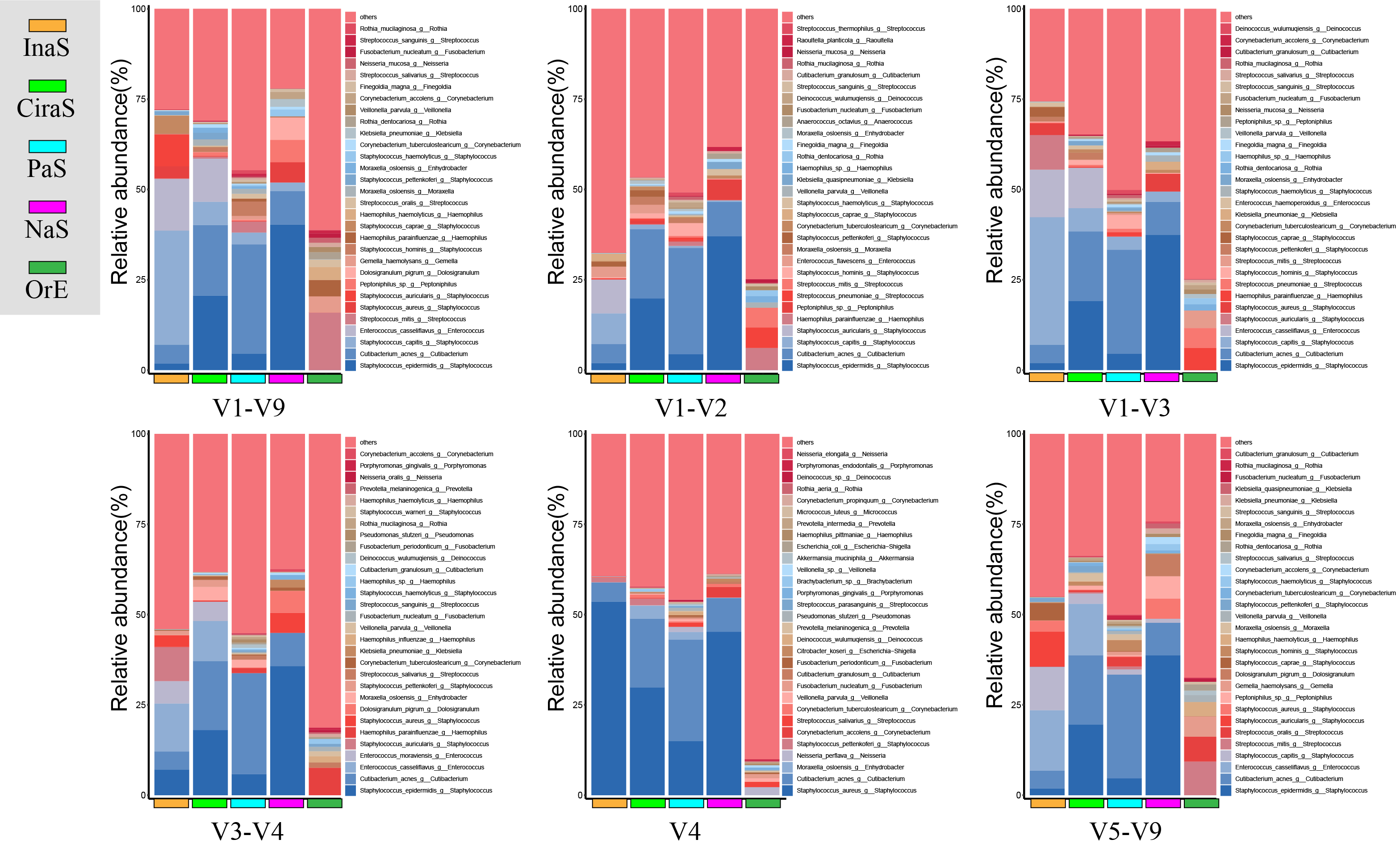

Supplement: Fig. S1 — Distribution of the top 30 high-abundance bacteria at the species level for various 16S regions. [file msystems.00399-24-s0001.tif]

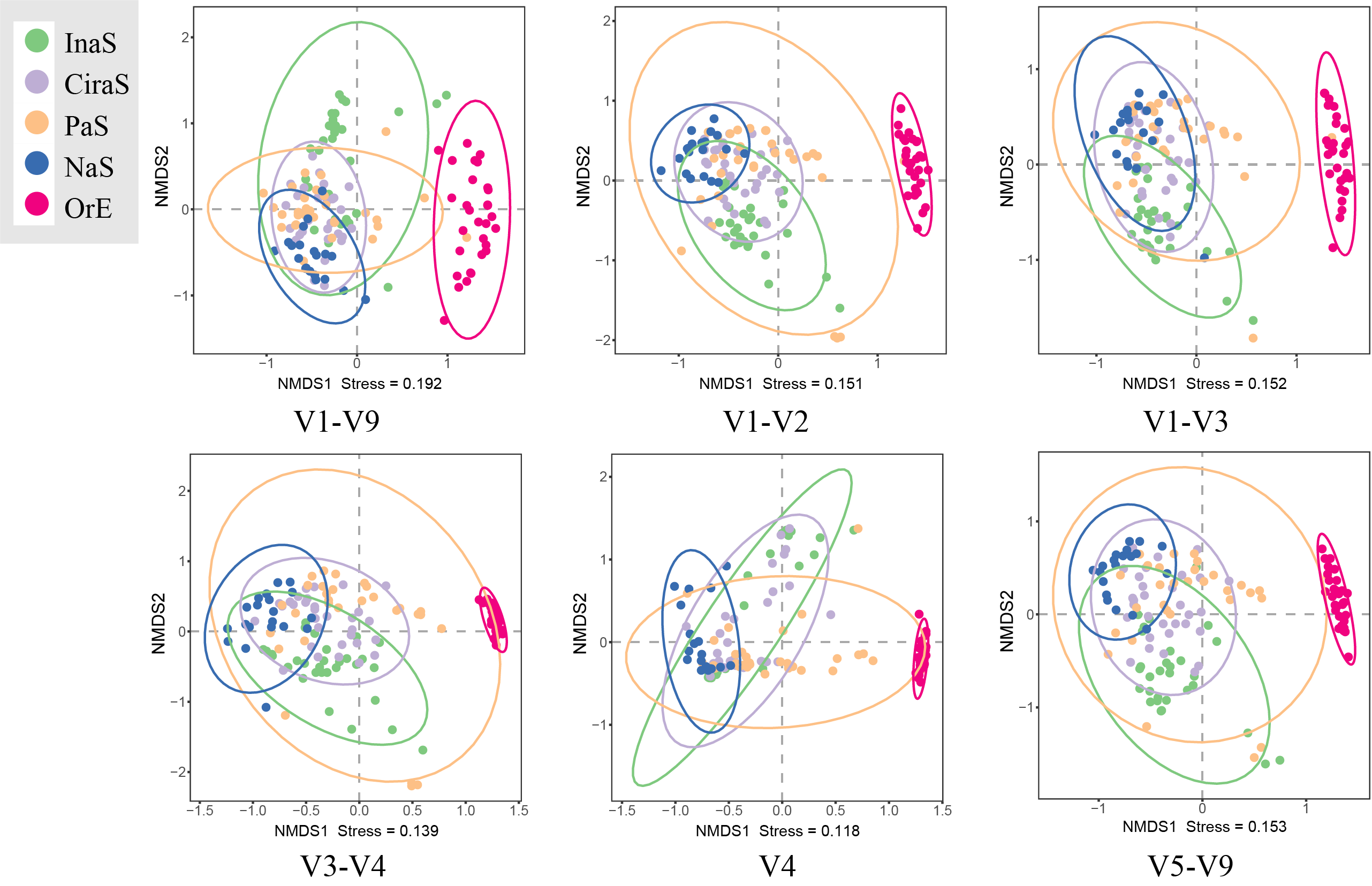

Supplement: Fig. S2 — Two-dimensional NMDS plot for different types of skin samples. [file msystems.00399-24-s0002.tif]

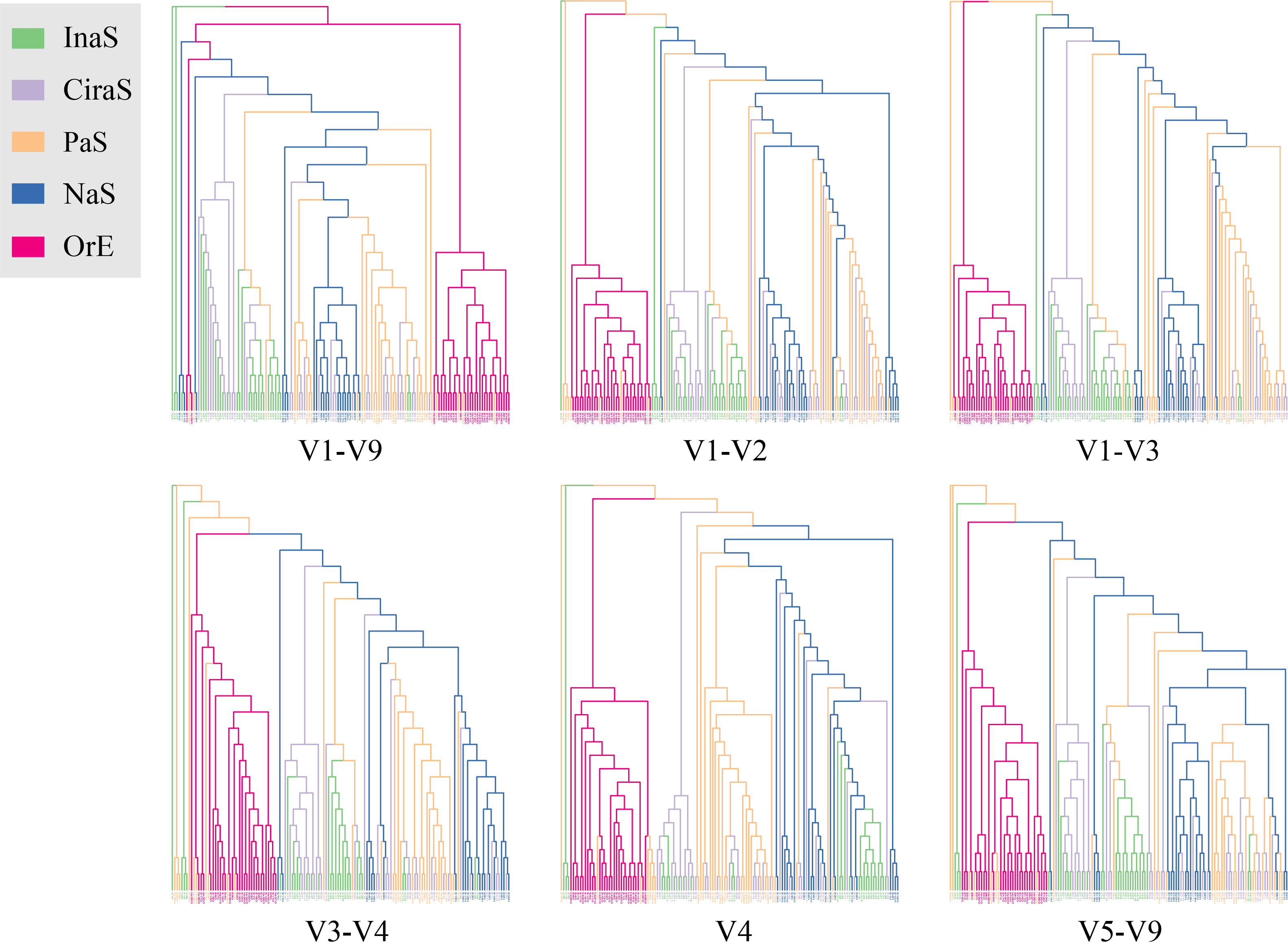

Supplement: Fig. S3 — Phylogram tree constructed from different types of skin samples. [file msystems.00399-24-s0003.tif]
