# Supplementary material for: Comparison of the full-length sequence and sub-regions of 16S rRNA gene for skin microbiome profiling
Source: mSystems. 2024 Jun 27;9(7):e00399-24. doi: 10.1128/msystems.00399-24 (PMC11264597; doi:10.1128/msystems.00399-24)
Supplement: Supplemental legends — Legends for Figures S1 to S3 and Tables S1 to S3. [file msystems.00399-24-s0004.docx]

**Supplementary Figure legends**

**Figure S1.** Distribution of the top 30 high-abundance bacteria at the species level for various 16S regions. The category labeled as “others” represents all low-abundance bacteria not depicted in the figure.

**Figure S2.** Two-dimensional nonmetric multi-dimensional scaling (NMDS) plot for different types of skin samples using the Bray-Curtis distance matrix.

**Figure S3.** Phylogram tree constructed from different types of skin samples based on the Bray-Curtis distance matrix.

**Supplementary Table legends**

**Table S1.** The primer pairs targeting the regions of the 16S rRNA gene were utilized in this study, including V1-V2, V1-V3, V3, V3-V4, V5-V9, and V1-V9.

**Table S2.** The microbial biomarkers and LDA values for different 16S regions across the five types of skin samples based on linear discriminant analysis effect size analysis (LEfSe). The bacteria identical to the microbial biomarkers selected from the V1-V9 region are shaded red, and the LDA values are shaded blue, with the width representing the magnitude of the values.

**Table S3.** The top 30 importance species and mean decrease Gini (MDG) values were selected by the random forest model. The shared bacteria with the V1-V9 region are shaded red, and the bacteria unique to each 16S sub-region are shaded white.
